# Comparison of Eylea® with Lucentis® as first-line therapy in patients with treatment-naïve neovascular age-related macular degeneration in real-life clinical practice: retrospective case-series analysis

**DOI:** 10.1186/s12886-015-0101-4

**Published:** 2015-08-20

**Authors:** Sophie C. Böhni, Mario Bittner, Jeremy P. Howell, Lucas M. Bachmann, Livia Faes, Martin K. Schmid

**Affiliations:** Eye Clinic, Cantonal Hospital of Lucerne, Spitalstrasse, 6000, Lucerne 16, Switzerland; Medignition Inc. Research Consultants, Zurich, Switzerland

## Abstract

**Background:**

To identify differences between Ranibizumab and Aflibercept in treatment-naïve patients with neovascular age-related macular degeneration (nvAMD) in a real-life clinical setting.

**Methods:**

We compared two groups of patients with a fairly similar prognosis either receiving Aflibercept or Ranibizumab within a pro re nata regimen for 1 year. Changes in visual acuity (letters) and central foveal thickness (CFT) and frequency of injections after completing the loading phase were evaluated using two separate multivariate mixed linear models.

**Results:**

When correcting for baseline differences between the Aflibercept (11 eyes) and Ranibizumab (16 eyes) group, there was neither divergence in visual acuity (−0.97 letters (95 % CI. −6.06-4.12); *p* = 0.709), nor a significant difference in the reduction of CFT (−25.16 μm, 95 % CI; (−78.01-27.68); *p* = 0.351) between the two groups 1 year after treatment initiation. Also, the number of injection did not differ (0.04 (95 % CI; −0.16-0.09); *p* = 0.565).

**Conclusion:**

In contrast to health claims, treatment-naïve nvAMD, Ranibizumab and Aflibercept were equivalent in terms of functional and morphologic outcomes and number of injections when studied in real-life clinical practice.

## Background

Due to its higher binding affinity and longer duration of action, Aflibercept (Eylea®, Regeneron, Tarrytown, New York, USA and Bayer HealthCare, Berlin, Germany) has theoretical advantages over Ranibizumab (Lucentis®, Gentech Inc., South San Francisco, CA, USA and Novartis AG, Basel Switzerland) [1, 2] in neovascular age-related macular degeneration (nvAMD) management, but empirical evidence quantifying the extent to which this translates into clinical practice is sparse. The recombinant fusion protein Aflibercept was licensed in October 2012 for treatment of nvAMD in Switzerland after the VIEW study [3, 4] showed its comparability in efficacy and safety to Ranibizumab. In contrast to Ranibizumab that only binds to VEGF-A [2, 5, 6], Aflibercept also binds to VEGF-B and the Placental Growth Factor, two additional factors of neovascularization [7, 8]. A mathematical model revealing a stronger binding affinity of Aflibercept to VEGF_165_ than Ranibizumab suggested that treatment intervals can be extended due to the longer duration of action [9].

However, even though clinical evidence that would allow for only bimonthly treatment with Aflibercept exists [3, 10], physicians often apply just the same ‘pro re nata’ (PRN) regimen with monthly check-ups and treatment if needed that was established for Ranibizumab in the PrONTO-studies [11, 12]. When applying the PRN regimen to Aflibercept during the second year of the VIEW-Trial [4], it was found that the new drug required fewer injections than Ranibizumab. But, a recent US American study, assessing the current use of Ranibizumab and Aflibercept in a real-life setting, found no differences regarding the therapeutic use of the two drugs [13]. Another recent health service research study corroborated these findings [14]. Unfortunately, both studies did not assess clinical outcomes associated with treatment. Therefore, in this 1-year retrospective analysis, we compared two groups of patients with treatment-naïve nvAMD and fairly similar prognosis either receiving Aflibercept or Ranibizumab at practitioner’s discretion and compared therapeutic use and corresponding clinical outcomes.

## Methods

A retrospective, comparative study of consecutive patients treated at the Eye Clinic of the Cantonal Hospital Lucerne (LUKS) over a 1 year period was conducted after seeking the approval of the ethics committee of Canton Lucerne. We retrospectively identified in our electronic medical records all patients who were started on either Ranibizumab or Aflibercept between 01.11.2012 and 31.12.2012 for the indication of newly diagnosed and therefore treatment-naïve nvAMD in at least one eye. Because of the retrospective nature of this study, no informed consent was obtained. We included patients with an observation period of at least 12 months, attending monthly follow-up visits where testing of best corrected visual acuity (BCVA) and optical coherence tomography (OCT) was performed For inclusion, patients needed the diagnosis of nvAMD secured by fluoresceine angiography beforehand. We included all patients with newly diagnosed and treatment-naive nvAMD. Since no information about the evolution of preliminary vision loss was available decisions for inclusion were not affected by this. All handwritten clinical records were manually searched for exclusion criteria. Exclusion criteria included: co-existence of visually significant ocular conditions (diabetic retinopathy, *n* = 1; non arteriitic anterior ischemic opticus neuropathy, *n* = 1), cataract surgery in the study eye within the year of follow-up (*n* = 4), YAG-capsulotomy in the study eye during follow-up (*n* = 0), conversion from one substance to another (Ranibizumab to Aflibercept, *n* = 6; Aflibercept to Ranibizumab, *n* = 1), insufficient clinical records (*n* = 1) and additional treatment for nvAMD (*n* = 0). Three patients were excluded for not receiving the initial loading dose and one patient was excluded because of intermediate discontinuation of treatment. When conditions stabilized, defined as absence of indication for intravitreal injection for at least 6 months, patients were henceforth cared for by their attending physician. This occurred in two patients, who were excluded from the analysis.

Patients suffering from cataract in the study eye were not primarily excluded, since this study is investigating the change in BCVA compared to baseline measurement. However, patients who underwent cataract surgery or - if already pseudophakic - a YAG-capsulotomy due to after-cataract during the time of follow-up in the study eye were excluded in order to prevent any biases arising from sudden gain in BCVA. The final study population consisted of 27 eyes of 24 patients, 16 eyes of 15 patients treated with Ranibizumab and 11 eyes of 9 patients treated with Aflibercept.

Whether patients were started on Ranibizumab or Aflibercept was decided at the practitioner’s discretion at first visit and under the assumption of similar efficacy as no proof of superiority for the one or the other substance in specific features of nvAMD was available. Therefore, the assignment was considered quasi-random, even more so as several practitioners were involved. All eyes included in this study had initially received a series of 3 monthly injections of either Ranibizumab or Aflibercept, afterwards treatment with both substances was continued in a PRN regimen. Retreatment criteria based on OCT included any existence of intraretinal or subretinal fluid, increase in retinal thickness >20um compared with the previous scan as well as increase of size of pigment epithelial detachment (PED) compared with the previous scan. Clinically, a newly detected macular haemorrhage in biomicroscopy accounted for the indication of retreatment. Futhermore, the visual development was carefully monitored and may have been taken into consideration in cases ambiguous by OCT.

Demographic data including patient age (at baseline) and gender, number of injections, BCVA and central foveal thickness (CFT) in OCT in included patients were explored (Table 1). We measured BCVA using the Early Treatment Diabetic Retinopathy Study (ETDRS) scale with best correction. Retinal images and automatic follow-up horizontal 13-line raster scans with automatically measured CFT were obtained with Spectralis-OCT (Heidelberg Engineering GmbH, 69121 Heidelberg, Germany) and evaluated with Heidelberg Eye Explorer (Version 1.7.1.0., ©2012, Heidelberg Engineering GmbH, 69121 Heidelberg, Germany). BCVA assessments as well as OCT measurements were conducted by experienced optometrists.

**Table 1:** Comparison of salient patients’ characteristics^a^ between the Ranibizumab and Aflibercept group at baseline, after completing the loading dose phase and after 1 year

	Ranibizumab (16 eyes)	Aflibercept (11 eyes)	Difference	Confidence interval	*p*-value
Baseline					
Age (mean ± SD) [years]	77.6 ± 9.20	75 ± 6.74	2.63	−4.08 to 9.33	0.428
Female gender n (%)	11 (68.8 %)	8 (72.7 %)			0.586
Right eye n (%)	7 (43.8 %)	6 (54.5 %)			0.436
Cataract n (%)	9 (56.3 %)	9 (81.8 %)			0.167
Serous PED n (%)	3 (18.8 %)	2 (18.2 %)			0.684
Classic CNV n (%)	7 (43.8 %)	3 (27.3 %)			0.324
Loading phase					
Letters before treatment (mean ± SD)	52.25 ± 23.38	62.55 ± 22.20	−10.30	−28.78 to 8.19	0.262
CFT before treatment (mean ± SD) [μm]	492.80 ± 165.60	352.50 ± 168.51	140.36	5.83 to 274.88	0.042
Letters after loading dose (mean ± SD)	66.06 ± 10.49	68.91 ± 8.51	−2.85	−10.71 to 5.01	0.463
CFT after loading dose (mean ± SD) [μm]	323.75 ± 93.00	267.73 ± 65.18	56.02	−10.93 to 122.98	0.097
1 year					
Letters after 1 year (mean ± SD)	67.25 ± 12.69	68.18 ± 13.06	−0.93	−11.29 to 9.43	0.856
CFT after 1 year (mean ± SD) [μm]	337.31 ± 110.14	274.00 ± 77.76	63.31	−16.13 to 142.75	0.113
Number of injections/year	8.28 ± 2.07	8.49 ± 1.97	−0.22	−1.85 to 1.42	0.787

^a^Descriptives were based on eyes

**Table 2:** Comparison of development of CFT and BCVA of the Ranibizumab and the Aflibercept group after the loading dose and after 1 year, respectively

	Ranibizumab (16 eyes)	Aflibercept (11 eyes)	Difference	Confidence interval	*p*-value
Loading phase					
Change of Letters after loading dose(mean ± SD)	13.81 ± 20.48	6.36 ± 15.46	7.45	−7.58 to 22.48	0.317
Change of CFT after loading dose (mean ± SD) [μm]	−169.06 ± 140.60	−84.73 ± 143.65	−84.34	−198.74 to 30.07	0.142
1 year					
Change of Letters after 1 year (mean ± SD)	15.00 ± 21.77	5.64 ± 12.64	9.36	−5.69 to 24.41	0.212
Change of CFT after 1 year (mean ± SD) [μm]	−155.50 ± 160.14	78.45 ± 165.83	−77.05	−208.08 to 53.99	0.237

Every OCT included in this study was manually revised and corrected if the automatic drawing of the inner limiting membrane and Bruch membrane necessary for measurement of the CFT were inaccurate. Furthermore, every patient’s nvAMD was classified in “predominantly classic” or “predominantly occult” on the basis of pre-therapeutic fluorescence angiography by an experienced retinologist.

Outcome was measured by change in BCVA and change in CFT measured by OCT after a treatment-period of 12 months with either substance compared to the values obtained after the loading dose, therefore 4 months after the initiation of treatment, which is consistent with the beginning of the PRN regimen. Furthermore, the number of injections needed in a PRN treatment regimen with the same retreatment criteria [11, 12] used for both substances was compared between the two groups.

### Statistical analyses

Sample size and power: We considered the two treatments to be equal if the difference in the average number of injections/year would be less than 10 % (non-inferiority margin). When enrolling 10 patients per treatment group and further assuming a standard deviation of this difference to be 0.5, the power would be 0.922.

We summarised continuous variables with means and standard deviations. Dichotomous variables are presented with percentages. Differences in population characteristics between patients receiving Ranibizumab and Aflibercept were tested using t-tests. A *p*-value of less than 5 % was considered as statistically significant.

Associations between changes in BCVA or CFT measured by OCT and type of treatment (Ranibizumab or Aflibercept) after completing the 3 month loading phase, were assessed with two multivariate mixed linear models. On one side, these models took into account that patients had provided repeated measurements during follow-up. To account for the fact that some patients provided data on both eyes, an indicator variate for “patient” was introduced as a random factor. Second, since differences in the distribution of parameters at baseline were present and therefore could introduce confounding, we accounted for these differences entering the two parameters with the largest differences between groups (letter values (continuous variate) and CFT values (continuous variate)) as additional parameters to the models.

Analyses were performed using the Stata 11.2 statistics software package. (StataCorp. 2009. Stata Statistical Software: Release 11. College Station, TX: StataCorp LP.)

## Results

Nine patients (11 eyes) with an average age of 75.0 years (SD 6.74) started with Aflibercept and 15 patients (16 eyes) with an average age of 77.6 years (SD 9.2) started with Ranibizumab. The proportion of female patients was similar between the two groups ((8/11) 72.7 % (Aflibercept) vs. (11/16) 68.8 % (Ranibizumab); 1-sided Fisher’s exact test *p* = 0.586). Further population characteristics are presented in Table 1. There were no documented SAEs in neither the ranibizumab nor the aflibercept group.

Patients receiving Aflibercept had a better average starting visual acuity compared to patients receiving Ranibizumab (difference 10.30 letters, standard deviation (SD): 8.98; *p* = 0.262). Moreover, the baseline CFT of patients receiving Aflibercept was significantly lower compared to patients on Ranibizumab (mean difference 140.4 μm (95 % CI; 5.83 to 274.88); *p* = 0.042).

When correcting for differences in BCVA at baseline between groups there was no difference within 1 year of follow-up ((−0.97 letters (95 % CI. −6.06 to 4.12); *p* = 0.709) with a mean letter value of 67.25 (SD 12.69) for ranibizumab and 68.18 (SD 13.06) for aflibercept. When correcting for these baseline differences, there was also no significant difference in the reduction of CFT between the two groups within 1 year (−25.16 μm, 95 % CI; (−78.01 to 27.68); *p* = 0.351). The number of injections was also similar (mean difference 0.04 (95 % CI; −0.16 to 0.09); *p* = 0.565), with an absolute number of 8.28 injections per 365 days (SD 2.07, CI 95 % 7.18 to 9.38) for ranibizumab and 8.49 injections/365 days (SD 1.97, CI 95 % 7.17 to 9.82) for patients under aflibercept treatment.

### Effect of loading dose on BCVA and CFT

For Ranibizumab, BCVA (mean difference 13.81 letters, (SD 6.41); *p* = 0.039) and CFT (mean difference 169.1 μm (SD 47.5); *p* = 0.001) improved significantly. In patients receiving Aflibercept these improvements were smaller and not significant (BCVA mean difference 6.36 letters, (SD 7.17); *p* = 0.386) and CFT (mean difference 84.7 μm (SD 54.5); *p* = 0.136).

### Effects during follow-up

After completing the loading phase, injections were associated with a small albeit non-significant improvement of letters 0.02 (95 % CI: −0.17 to 0.22) *p* = 0.802) There was also a non-significant increase of CFT after 1 year in all patients (+56.09 (95 % CI: −197.95 to 310.13) *p* = 0.647) which was also similar between the two patient groups. Changes of letters and CFT after completing the loading phase and 1 year are shown in Table 2. 

## Discussion

### Main findings

In this retrospective real-life clinical assessment of previously untreated patients with nvAMD, Aflibercept being featured with longer duration of action and stronger binding affinity showed no advantages over Ranibizumab when treating for one-year. As first-line treatments of nvAMD, both substances were equivalent in terms of injection frequency and visual outcome. Moreover, no statistically significant differences were found between CFT changes along 1 year of follow-up between both treatment groups.

### Implications for research

Basic scientific enquiry showed that Aflibercept has a binding affinity to various types of VEGF and also to Placental Growth Factor resulting in higher efficiency [3, 4]. Based on these findings advantages of Aflibercept over Ranibizumab in clinical efficacy have been proposed [4, 9]. Our data, collected in the real-life clinical setting, do not confirm these results. Why not, remains incompletely understood and calls for further research. It can be speculated that the research protocols for the Ranibizumab treatment in the VIEW studies led to additional injections that are considered unnecessary in clinical practice. As Aflibercept poses the ‘newbie’ in the group of anti-VEGF-substances, further real-life data is yet to be collected. Further research should also explore ophthalmologists’ attitudes towards anti-VEGF treatments. In our study, the decision whether to start with Aflibercept or Ranibizumab was fully at the discretion of the treating physician and we expected that equipoise was present. Nevertheless we observed considerable differences of baseline BCVA and CFT between the two groups indicating that they applied some sort of tacit algorithm. A better understanding of criteria guiding ophthalmologists’ decisions could help depicting accepted management principles.

### Our findings in context of Literature

To our knowledge, this is the first study comparing Ranibizmab’s and Aflibercept’s efficacy in a real-life setting. We are aware of one recent study by Johnston and colleagues who counted the injection frequency and summarized the expenditures of Ranibizumab and Aflibercept treatments using health insurance claims data from the United States [13]. Concordant to our results, they found no statistical differences between the two drugs.

### Strengths and limitations

The strength of this study lies in the early evaluation of this topic as Swiss authorities approved Eylea only in November 2012. Therefore it will contribute to a better understanding of Aflibercept’s role in a real-life clinical setting. Studies like ours have the potential to quantify effects of treatments as they occur in clinical practice. This has many reasons. Among them, the selection of patients receiving care is not as rigorous as it usually is in controlled experiments. Moreover, even in a highly standardized clinical environment, the way of patient work-up and follow-up is less intensive as in many studies but follows the laws of standard care. Both aspects bear the potential of larger effects in clinical trials as possibly observed in daily clinical routine. As a limitation one must consider the non-randomised nature of the study, where selection bias cannot unerringly be excluded. Actually, we found differences in the baseline characteristics between the two groups in terms of visual acuity and central foveal thickness indicating that the experienced ophthalmologists enrolled in this study applied some tacit selection mechanism. However, even though a clinical protocol can never be replaced, the study was conducted with a rigorous work-up protocol. Moreover, the analysis corrected for different baseline values and also excluded the measurements of the loading phase to make groups more comparable. Finally, the study was of limited size and thus, estimates were imprecise warranting further confirmation in new studies.

### Implications for practice

This study provided anti-VEGF drugs according to the popular PRN approach. Our results, thus, are particularly useful for all those retinologists using this regimen. The retrospective analysis made in this cohort of patients did not detect significant differences between ranibizumab and aflibercept suggesting that both drugs could be used as first line therapies. Given that the price for both substances is identical (CHF 1067.-/dose) [15], preferential use of one substance for economic reasons - as could be the case with Bevacizumab and Ranibizumab - might play no role in the competition between Ranibizumab and Aflibercept. We presume that further practical experience and research will allow defining the clinical niches where the two drugs show their optimal efficacy profile. Latest evidence [16–23] was able to prove Aflibercept’s utility as a ‘salvage therapy’ in cases of suspected tachyphylaxis [24–26] to Ranibizumab, with gain in visual acuity and/or reduction of CFT after conversion. Furthermore, clinical observation and results from case reports suggest that patients with serous PED might benefit from treatment with Aflibercept [27, 28]. But this observation yet needs to be backed up with experimental clinical data.

## Conclusion

The claimed advantages of Aflibercept, the new concurrent to Ranibizumab in nvAMD management, did not materialize in this one-year clinical analysis under real-life circumstances. The present study suggests that both drugs are equivalent in terms of visual and anatomical outcomes. However, further well designed comparative studies investigating the two drugs in a PRN treatment regimen evaluating the potential clinical advantages of Aflibercept over Ranibizumab as first-line therapy in patients with nvAMD should be conducted.

## Abbreviations

nvAMD: Neovascular age-related macular degeneration
CFT: Central foveal thickness
PRN: Pro re nata
LUKS: Eye Clinic of the Cantonal Hospital Lucerne
BCVA: Best corrected visual acuity
OCT: Optical coherence tomography
PED: Pigment epithelial detachment
ETDRS: Early treatment diabetic retinopathy study
